# Hemodynamics and Function of Resistance Arteries in Healthy Persons and End Stage Renal Disease Patients

**DOI:** 10.1371/journal.pone.0094638

**Published:** 2014-04-10

**Authors:** Morten K. Borg, Per Ivarsen, Emil Brøndum, Johan V. Povlsen, Christian Aalkjær

**Affiliations:** 1 Department of Biomedicine, Aarhus University, Aarhus, Denmark; 2 Department of Renal Medicine, Aarhus University Hospital and Department of Clinical Medicine, Faculty of Health, Aarhus University, Aarhus, Denmark; University of Sao Paulo Medical School, Brazil

## Abstract

**Introduction:**

Cardiovascular disease is the leading cause of death in patients with end stage renal disease (ESRD). The vasodilator mechanisms in small resistance arteries are in earlier studies shown to be reduced in patients with end stage renal disease. We studied whether endothelium dependent vasodilatation were diminished in ESRD patients and the interaction between the macro- and microcirculation.

**Methods:**

Eleven patients with ESRD had prior to renal transplant or insertion of peritoneal dialysis catheter measured pulse wave velocity. During surgery, a subcutaneous fat biopsy was extracted. Resistance arteries were then dissected and mounted on a wire myograph for measurements of dilator response to increasing concentrations of acetylcholine after preconstriction with noradrenaline. Twelve healthy kidney donors served as controls.

**Results:**

Systolic blood pressure was elevated in patients compared to the healthy controls; no difference in the concentration of asymmetric dimethyl arginine was seen. No significant difference in the endothelium dependent vasodilatation between patients and controls was found. Correlation of small artery properties showed an inverse relationship between diastolic blood pressure and nitric oxide dependent vasodilatation in controls. Pulse pressure was positively correlated to the total endothelial vasodilatation in patients. A negative association between S-phosphate and endothelial derived hyperpolarisation-like vasodilatation was seen in resistance arteries from controls.

**Conclusion:**

This study finds similar vasodilator properties in kidney patients and controls. However, correlations of pulse pressure and diastolic blood pressure with resistance artery function indicate compensating measures in the microcirculation during end stage renal disease.

## Introduction

Cardiovascular disease (CVD) is overrepresented in patients with reduced kidney function, and the risk increases with declining kidney function, why CVD is the major cause of death in patients with end stage renal disease (ESRD) [1]. Both arterial stiffness and small artery structural and functional alterations are involved in the pathophysiology leading to changes in the cardiovascular system, but the mechanisms responsible are not fully elucidated.

Arterial stiffness, defined as decreased ability and degree of the conductive arteries to absorb the pulse pressure is related to mortality in dialysis and non-dialysis patients [2], [3]. The degree of arterial stiffness can be estimated by measuring pulse pressure, pulse wave velocity (PWV) or augmentation index (AI). The mechanisms inducing stiffening of the conductive arteries are complex and include both non-renal risk factors e.g. smoking, diabetes, hypertension and renal risk factors such as calcification, increased plasma concentration of asymmetric dimethylarginine (ADMA), inflammation and oxidative stress [4], [5].

The function of the large arteries' viscoelasticity reduces the pulsative pressure and flow that results from the intermittent ventricular ejection, securing a stabile pressure and flow at the level of small arteries. When stiffness increases, an augmentation of the pulse wave is present because of changes in the timing and size of reflection. This might induce damage and change the functionality of the vascular microcirculation as it is exposed to a higher pulsative pressure and flow.

Endothelium-dependent vasodilatation occurs via three main pathways; cyclooxygenase (COX) products, nitric oxide (NO) and endothelium-derived hyperpolarisation (EDH) [6]. Endothelium-dependent vasodilatation in the large arteries from ESRD patients has been examined with forearm blood flow measurements and shown to be associated with an impairment of the vasodilator properties due to a defect in the NO-pathway [7]. Similar results have been obtained in brachial artery, where shear stress induced vasodilatation in ESRD patients was found attenuated at maximal shear stress [8]. The diminished vasodilator function in the macro circulation of ESRD patients, measured as post-ischemic reactive hyperemia, is associated with increased all-cause mortality [9].

Investigations of the micro circulation (the resistance arteries) of ESRD patients show no difference from similar arteries from healthy controls when comparing morphology and sensitivity to vasoconstrictors [10]. Endothelium independent vasodilatation is also found unchanged, whereas studies examining the endothelium dependent vasodilatation have yielded different results, as resistance arteries from ESRD patients have shown to have a reduced relaxation to acetylcholine (ACh) [11]. This appears to be due to a defect in the NO-pathway [12], while another study shows that the EDHF-response is attenuated, but only when using bradykinin as the agonist and not ACh [13].

In this study we wanted to evaluate differences in vascular function in arteries from patients with ESRD and normal controls and to study the interaction between the macro- and micro-circulation. Especially we wanted to test whether endothelium dependent vasodilatation was diminished in ESRD patients with well controlled blood pressure.

## Materials and Methods

### Study population

Eleven patients with ESRD were enrolled in the study. Nine had living related donor renal transplant and two insertion of peritoneal dialysis catheter. Patients included were above 18 years of age. Exclusion criteria were persisting cardiac arrhythmias, severe congestive heart failure, reduced pulmonary function, severe psychiatric disease, acute infection and leg-amputation. Five patients were treated with peritoneal dialysis (vintage 252 days (101-568) median (range)); four with hemodialysis (vintage 769 days (14-1201)) and two were not on dialysis. Patient's kidney diseases were glomerulonephritis (n = 5), adult polycystic kidney disease (n = 2), obstructive nephropathy (n = 2), type 1 diabetes mellitus (n = 1) and vasculitis (n = 1). No patient had diabetes mellitus as comorbidity. Nine out of eleven were treated with antihypertensive medication. Two were treated with angiotensin converting enzyme inhibitors, four with angiotensin receptor blockade, six with calcium antagonists, four with beta-blockers, six with diuretics, ten with erythropoietin analogs and one with statins.

The majority of patients were cardiovascular assessed as part of the pre-transplantation assessment or ESRD regular control with echocardiography, coronary angiogram and CT scans of the iliac arteries. All investigation was performed in the year before the study and none had any cardiovascular event between the cardiovascular assessment and transplantation. All patients had an echocardiography; ten had normal ejection fraction (EF) while one patient had an EF of 40%. One of the patients had indication of a light aortastenosis and five patients had light left ventricular hypertrophy. Nine patients had a coronary angiogram; seven patients had normal coronary arteries, one had earlier coronary by-pass and one had some calcification but no stenosis. Seven had a CT scan of the iliac arteries; five had no calcification, 2 had mild to moderate calcification. Cholesterol was measured in six patients, with a mean value of 4.9 mmol/l. Inflammatory profile were assessed with c-reactive protein in ten of eleven patients prior to surgery; all values were less than 10 mg/l.

Twelve kidney donors with no preexisting medical conditions or drug intake served as healthy controls. Before surgery the kidney recipients received 2 days of immunosuppressive therapy consisting of 0.2 mg/kg tacrolimus, 1.5 g mycophenolat mofetil acid and 20 mg prednisolone. The study was performed in accordance with the Declaration of Helsinki. Protocol and consent forms were approved by the local research Ethics Committee (Central Denmark Region), and all participants gave written informed consent before inclusion.

### Protocol

Participant's body weight, height and blood pressure were measured and hemodynamic data were calculated the day before surgery. Patient's type of dialysis, vintage, underlying condition and drug intake were obtained from medical records. Blood samples were taken on the day of surgery. During surgery a 2×3 cm biopsy containing skin and subcutaneous fat was removed from the abdominal wall with scalpel without the use of diathermy.

### Microvascular function

Subcutaneous fat biopsies were immediately after extraction placed in 5°C physiological salt solution (PSS), transported to the lab where 2 mm long segments of arteries were isolated and mounted on two stainless steel wires (40 μm diameter) in the organ baths of a 4-channel wire myograph (model 610M, Danish Myo Technology (DMT), Aarhus, Denmark) or in a double channel myograph (model 410A, DMT) for isometric force measurement. The myograph contained PSS at 37°C, continuously bubbled with a gas mixture containing 5% CO_2_and 21% O_2_ in a nitrogen based gas to keep pH at 7.4 at all times. Upon mounting, the arteries were left 20 min to equilibrate before stepwise stretching, characterizing the elastic properties as described by Mulvany and Halpern [14]. Experiments were conducted at 90% of L_100_ (defined as the circumference of the relaxed artery exposed to a transmural pressure of 100 mmHg). Viability of the arteries was tested using 10 μM noradrenaline (NA) twice before beginning the experiments. The arteries were preconstricted with 3 μM NA and subsequent relaxation using increasing concentrations of the endothelium-dependent vasodilator ACh. The experiment were repeated after incubation (20 min) with first the COX-inhibitor indomethacin (3 μM) and then the combination of the NOS inhibitor L-NAME (100 μM) and indomethacin.

To ensure that the endothelium was viable no artery was submitted to more than 3 endothelium-dependent relaxation curves and were left resting for 20 minutes between curves during incubation.

The composition of PSS was (mM): NaCl 119, KCl 4.7, KH_2_PO_4_ 1.18, MgSO_4_ 1.17, NaHCO_3_ 25, CaCl_2_ 1.6, EDTA 0.026, and glucose 5.5. The chemicals were obtained from Sigma (St.Louis, MO, USA). Indomethacin was dissolved in ethanol, the other chemicals in distilled water.

### Hemodynamics

Cardiac output (CO) was measured by rebreathing technique in a closed system containing a gas mixture of sulfahexafloride and nitrous oxide in a mixture of oxygen and nitrogen (Innocor, Denmark). Rebreathing was performed in the sitting position during 15 s with a breathing rate of 14–16 min^−1^ and a volume of 1.8 l after a rest of at least 10 min [15]. Gas was sampled continuously from the mouthpiece and analyzed online on an infrared gas analyzer. Pulmonary blood flow (PBF) was calculated from uptake rate of nitrous oxide into the blood. The first two or three breaths were excluded from analysis if the total lung volume measured by sulfahexafloride indicated incomplete gas mixture. In the majority of patients without pulmonary arterio-venous shunt PBF equals CO [16], [17]. In contrast in patients with pulmonary shunt, the shunt fraction is calculated and added to PBF to get CO. The shunt fraction is calculated from the oxygen concentration [18]. The calculations were performed assuming that the gasses were mixed completely and that the equilibration of gasses between alveoli and capillary was rapid and that lung flow was constant. Systolic blood pressure (SBP) and diastolic blood pressure (DBP) were measured by an automatic device connected to the Innocor. Systemic vascular resistance index (SVRi) was calculated as: (Mean arterial blood pressure – central venous pressure)/Cardiac output and indexed to body surface by Innocor. The measurement was performed twice, and the mean values were used in data analysis.

PWV and AI were measured in the supine position after 10 min of rest. Carotid –femoral PWV was measured with SphygmoCor, AtCor Medical, Texas, US, using the integral software.

Augmentation pressure was calculated as the difference between the second and first systolic peaks, and AI was calculated as the augmentation pressure expressed as percentage of pulse pressure. AI was measured for aorta. All of the measurements were made in duplicate by one trained study nurse, and the mean values were used in the subsequent analysis.

### Biochemistry

Asymmetric dimethyl arginine (ADMA) was measured using ELISA (DLD Diagnostika GmbH, Germany). All other biochemistry was analysed at the Department of Clinical Biochemistry, Aarhus University Hospital. EGFR was calculated from the 4 point MDRD formula.

### Statistics

Data in figures are presented as mean ± SEM, in tables as mean ± SEM or median (range). Concentration-response curves to ACh are given as percentage relaxation of the pre-constriction to NA. EC_50_ values are calculated by non-linear regression for variable slope for each vessel and presented as the negative logarithmic value in mol/L (pEC50). NO dependent response was calculated as the difference between EC_50_ values of indomethacin and indomethacin/L-NAME curves respectively (ΔpEC_50_). Mean pEC_50_, ΔpEC_50_ and baseline characteristics on patients were compared using unpaired student's *t*-test. Associations between variables were calculated using Pearson's correlation. P<0.05 were considered significant. All statistical analyses were performed with GraphPad Prism (v. 4.03 GraphPad Software Inc., CA, US)

## Results

### Study population

Kidney function was significant lower in ESRD patient, as expected. Age, weight and BMI were similar, whereas systolic, but not diastolic, blood pressure was significantly elevated in ESRD patients (Table 1), despite antihypertensive treatment. Blood hemoglobin concentration was decreased in ESRD patients, whereas no significant difference in ADMA concentration was found.

**Table 1:** Baseline characteristics for end stage renal disease (ESRD) patients and healthy controls.

Variable	ESRD (n = 11)	Controls (n = 12)
**Sex (male/female)**	6/5	4/8
**Age (years)**	51(19–85)	54(36–70)
**S-Creatinine (μmol/mL)**	806±82*	68±3
**eGFR (mL/min/1,73 m^2^)**	6.7±0.7*	87.5±4.2
**Systolic blood pressure (mmHg)**	130±2*^§^	119±4^#^
**Diastolic blood pressure (mmHg)**	78±3^§^	76±2^#^
**Pulse pressure (mmHg)**	52±3^§^	43±4^#^
**Weight (kg)**	73.2±3.2^§^	75.9±3.8^#^
**BMI (kg/m^2^)**	24.6±1.2^§^	26.2±0.9^#^
**Hemoglobin (mmol/L)**	7.2±0.3*	8.2±0.2
**S-Calcium-ion (mmol/L)**	1.18±0.03	1.22±0.02
**S-Phosphate (mmol/l)**	1.87±0.10*	1.14±0.03
**S-PTH (pmol/l)**	24.9±5.1	NA
**Albuminuria (g/day)**	3.38±1.30*^§^	0±0
**ADMA (μmol/L)**	0.78±0.04^§^	0.72±0.02

Data are mean±SEM, except for age which is median (range); * p<0.05, ^§^ n = 10, ^#^ n = 11.

### Microvascular function

**Figure 1:**
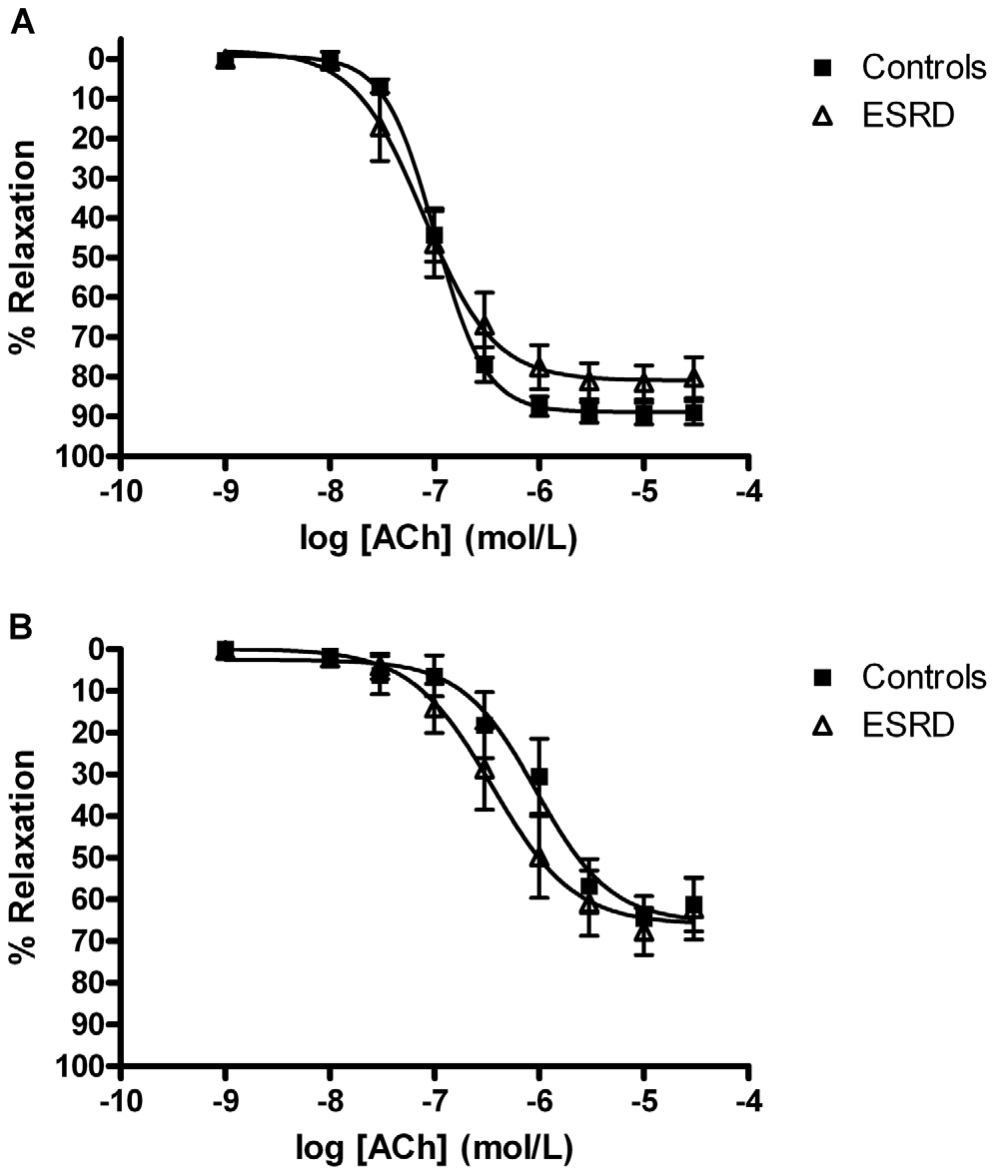
Concentration-response relaxation curves to acetylcholine in arteries from healthy controls and patients with end stage renal disease. A: Endothelium dependent vasodilatation; p = ns. B: Endothelium-derived hyperpolarisation-like relaxation in the presence of indomethacin and L-NAME; p = ns.

Relaxation of subcutaneous resistance arteries with ACh was not significantly different between ESRD patients and controls (Figure 1A). Both maximal relaxation and pEC50 were comparable (maximal relaxation: 84±4% vs. 90±2%, p = 0.22; pEC50: 7.06±0.12 vs. 6.97±0.06, p = 0.52).

The EDH-like relaxation was recorded after blockade of eNOS and COX (Figure 1B). The maximal relaxation and sensitivity to ACh was similar between the two groups (maximal relaxation: 68±5% vs. 66±5%, p = 0.82; pEC50: 6.39±0.17 vs. 6.15±0.21, p = 0.38).

**Figure 2:**
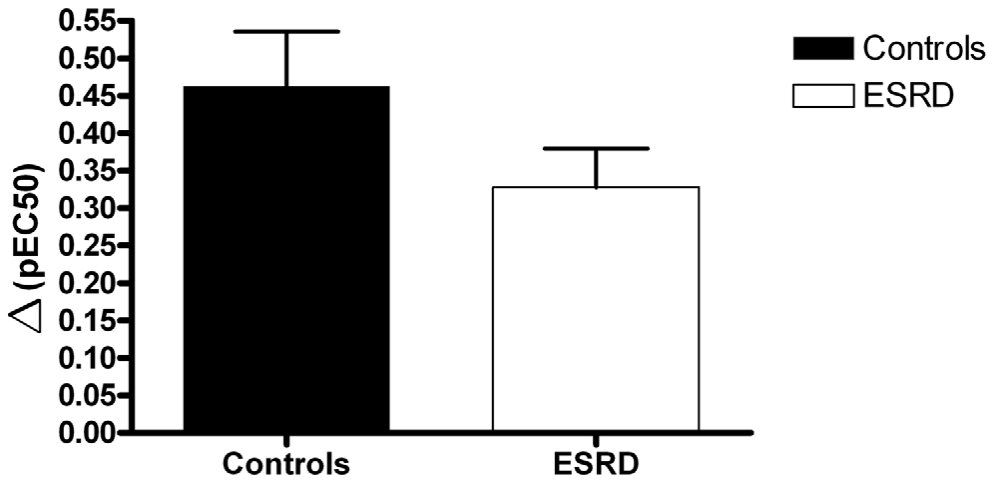
Nitric oxide dependent-response, calculated as the difference between pEC_50_-values of concentration response curves incubated with indomethacin and indomethacin/L-NAME respectively – ΔpEC_50_; controls (n = 10), ESRD (n = 11); p = 0.15.

The NO-dependent vasodilatation was calculated as ΔpEC_50_ for concentration response curves incubated with indomethacin and indomethacin + L-NAME respectively (figure 2). No significant difference between ESRD and controls was found (ΔpEC_50_ was 0.33±0.05 vs. 0.46±0.07, ESRD and controls, respectively, p = 0.15).

**Figure 3:**
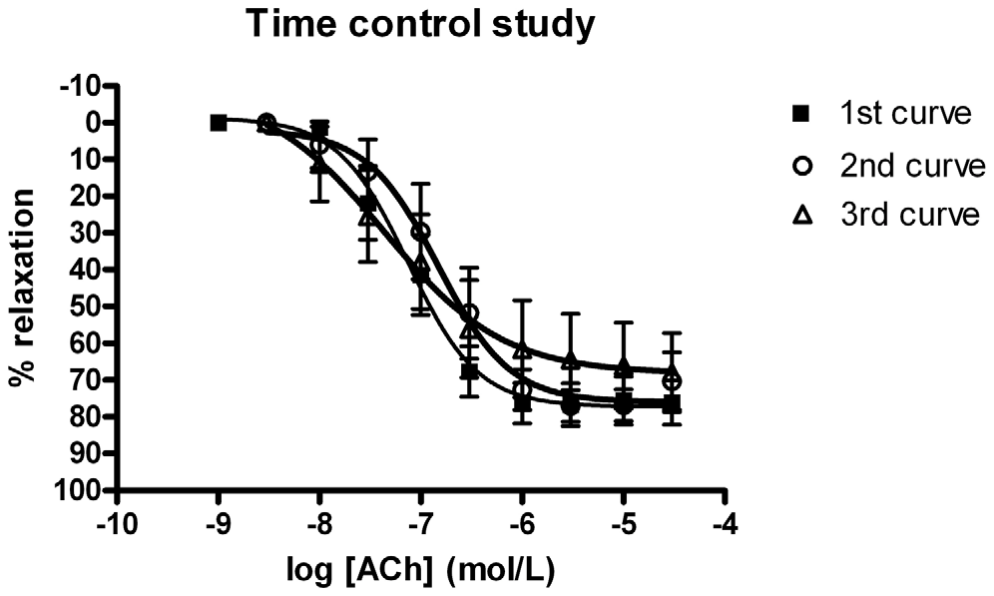
Time control experiments; three consecutive concentration response curves, with 20 minutes of rest in between, without use of blocker.

To assess whether three concentration response curves to acetylcholine can be obtained, time control experiments were performed. Three concentration response curves, with 20 minutes of rest in between, were conducted; without use of any blocking agent (figure 3). As seen there was no deterioration in the response to acetylcholine with time.

### Hemodynamics

PWV and SVRi were not significantly different in the two groups, while AI was lower in the ESRD group (table 2), which was not expected. This was probably because of small sample size.

**Table 2:** Hemodynamics in patients with end stage renal disease (ESRD) and healthy controls.

Variable	ESRD (n = 5)	Controls (n = 12)
**Pulse wave velocity (m/s)**	11.38±3.33^§^	7.11±0.39^#^
**Augmentation index (%)**	122±7*	154±9^#^
**Cardiac output index (L/min/m^2^)**	3.58±0.42*	2.79±0.14
**SVR index (mmHg/(L/min)/m^2^)**	28.05±2.99	32.58±1.40

Systemic vascular resistance (SVR).

Data are mean±SEM; * p<0.05; ^§^ n = 4; ^#^ n = 10.

### Associations between micro- and macrovascular functions

**Figure 4:**
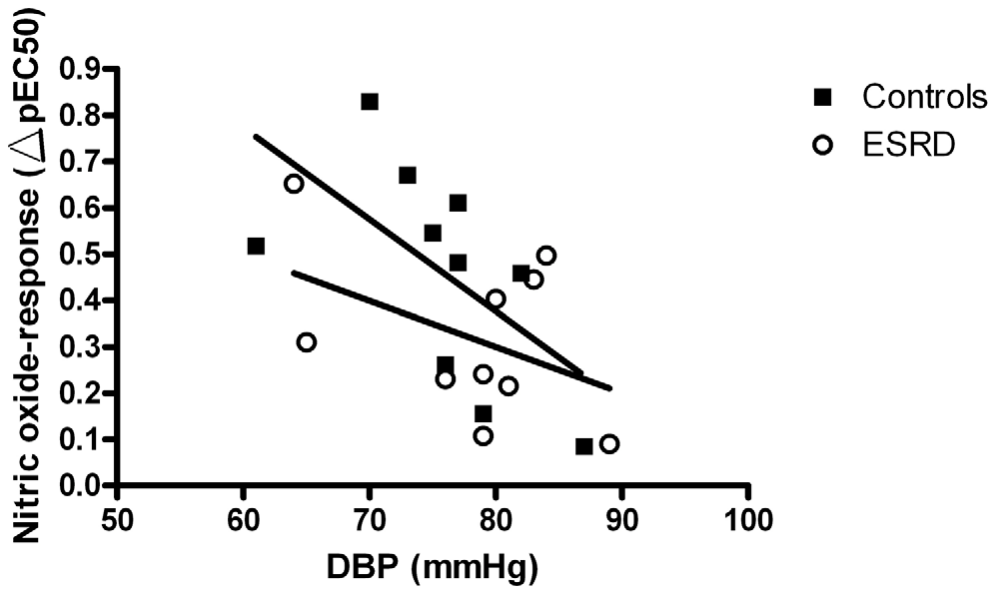
Association between diastolic blood pressure and nitric oxide-response (ΔpEC_50_) in controls (r = −0.59; p<0.05) and ESRD (r = −0.44; p = 0.10), Pearson one-tailed correlation.

DBP was inversely related to the NO-dependent vasorelaxation in controls (r = −0.59; p<0.05) (figure 4). The inverse relation was not significant in ESRD (r = −0.44; p = 0.10).

**Figure 5:**
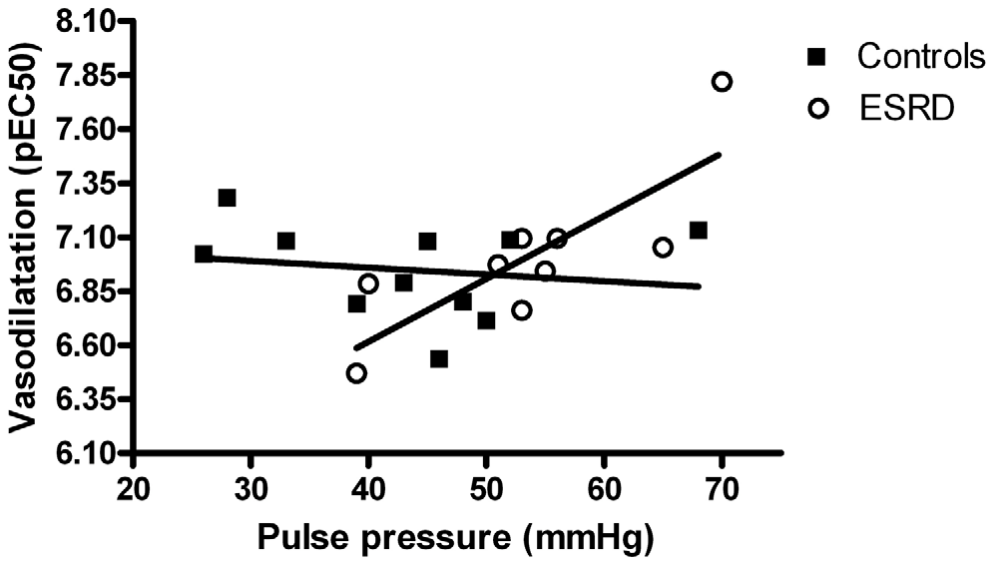
Association between pulse pressure and vasodilatation (pEC_50_) in controls (r = −0.17; p = 0.31) and ESRD (r = 0.81; p<0.01), Pearson one-tailed correlation.

Pulse pressure was positively correlated to ACh induced vasodilatation in ESRD (r = 0.81; p<0.01), this was not the case in control subjects (r = −0.17; p = 0.31) (figure 5).

**Figure 6:**
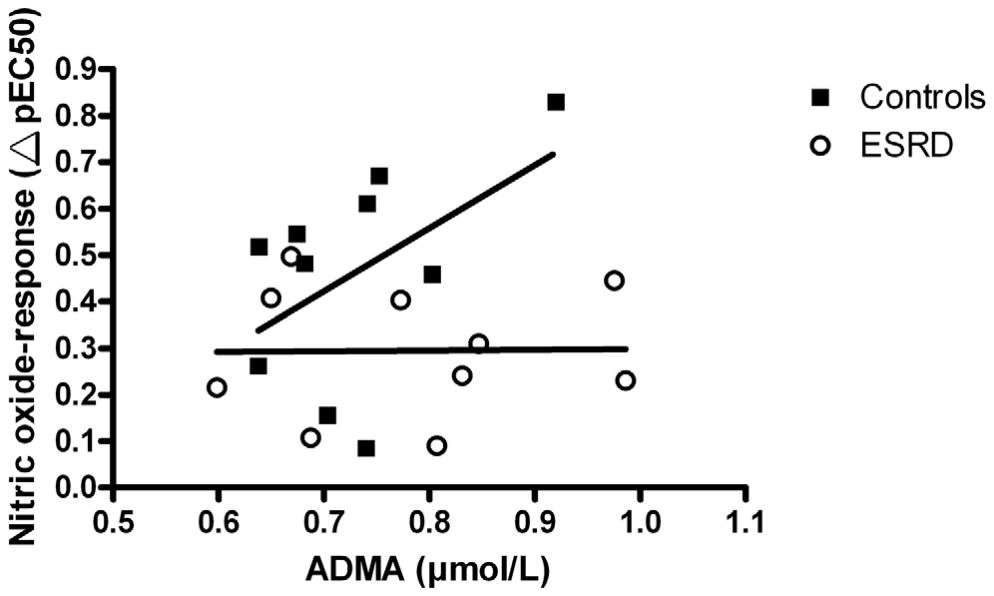
Association between ADMA and NOS dependent relaxation (ΔpEC_50_) in controls (r = 0.49<; p = 0.07) and ESRD (r = 0.01; p = 0.97), Pearson one-tailed correlation.

Correlation of ADMA and NO-response showed a trend in controls (figure 6), although not statistically significant (r = 0.49; p = 0.07), whereas no trend was seen in ESRD (r = 0.01; p = 0.97).

**Figure 7:**
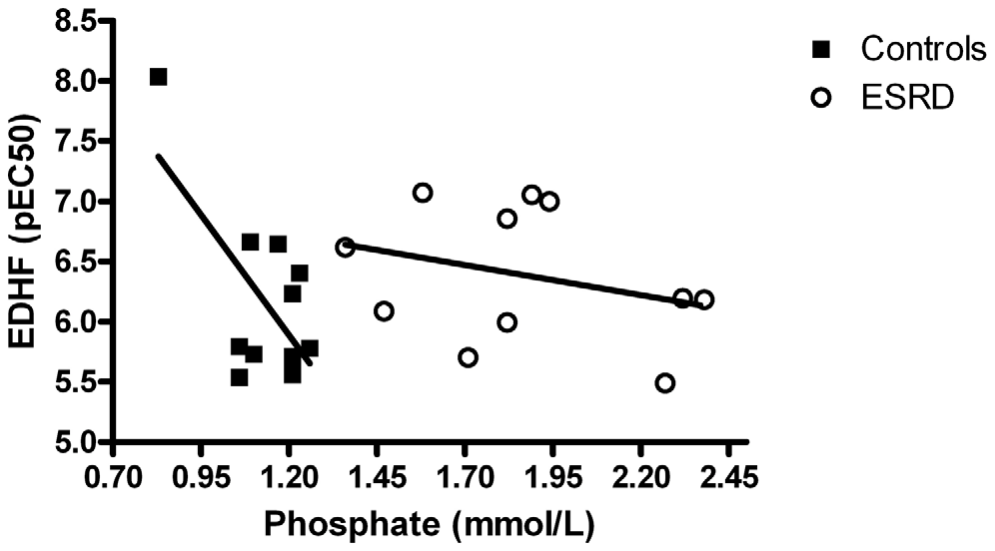
Association between S-Phosphate and EDH-like relaxation (pEC_50_) in controls (r = −0.66; p<0.05) and ESRD (r = −0.30; p = 0.18), Pearson one-tailed correlation.

As expected S-phosphate levels were different in the two groups. A negative association was found between S-phosphate and EDH-like response in controls(r = −0.66; p<0.05), but not in ESRD (r = −0.30; p = 0.18) (figure 7).

**Figure 8:**
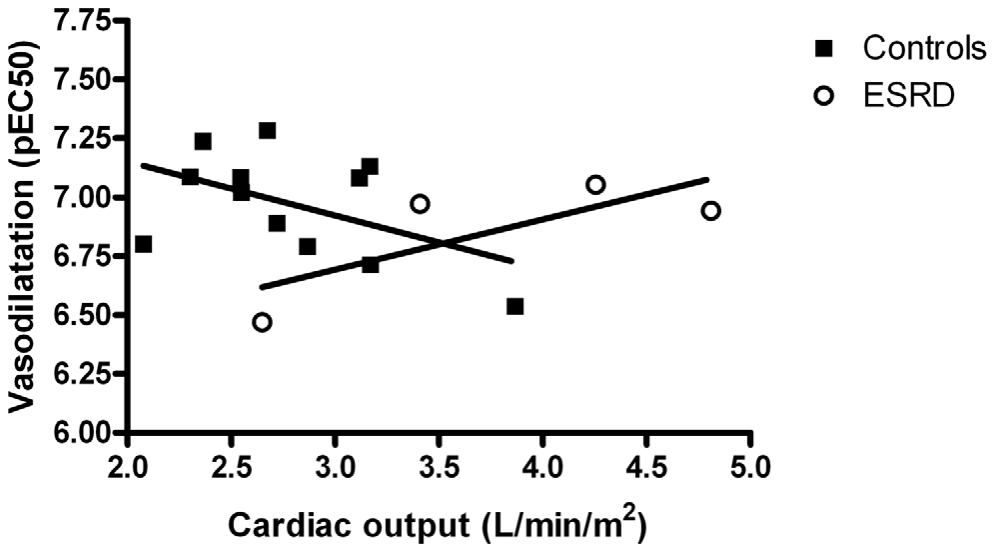
Association between cardiac output and vasodilatation (pEC_50_) in controls (r = −0.50; p = 0.05) and ESRD (r = 0.76; p = 0.12), Pearson one-tailed correlation.

Cardiac output and vasodilatation were inversely related in controls (figure 8), higher cardiac output resulted in a diminished vasorelaxation (r = −0.50; p = 0.05). While no relation was present in ESRD (r = 0.76; p = 0.12).

## Discussion

In the present study, we evaluated micro- and macrovascular differences in ESRD patients and healthy controls and for the first time correlated these findings. The ESRD patients had little comorbidity and well controlled blood pressure. In the microvascular function no significant different response to endothelium dependent vasodilatation was seen between ESRD and healthy controls. In the macrovascular arteries a significant higher SBP was present in the ESRD patients. Micro- and macrovascular function was correlated, DBP and the NO-dependent vasorelaxation was inversely related in controls. Pulse pressure was positively correlated to endothelial vasodilatation in ESRD and interestingly a negative correlation between S-phosphate and EDH-like response was found in controls.

The microvascular endothelial dependent vasodilator response to ACh has earlier been shown to be diminished in ESRD, while attempts to determine the affected pathway, has yielded diverging results [11]–[13]. In our study a similar vasodilator response to ACh was found, both under control conditions and after inhibition of NOS and COX dependent pathways. The earlier studies suggested that the ACh induced vasodilatation is diminished in ESRD patients [11]; others have confirmed this and suggested that the affected pathway is NOS dependent [12] or involves EDH-like relaxation [13]. Luksha et al. [13] found that only bradykinin-induced EDH-like relaxation was reduced, while ACh-induced EDH-like relaxation was similar in the two groups. Hence parts of our results differ from earlier studies. The diverging results must rely on a complex mixture of a different period of exposure to the uremic milieu, patients' age, physical form, pharmacological treatment, dialysis and blood pressure levels while the experimental setup seems comparable. The lack of a significant difference in resistance artery function in the two groups is consistent with the similar SVRi in the two groups.

Our patients have substantial lower blood pressure than the patients in the aforementioned papers. Since diastolic hypertension is known to cause an attenuated ACh induced vasorelaxation in the resistance arteries [19], the possibility of normal endothelial function in resistance arteries of ESRD patients with well controlled blood pressures seems feasible. We found a negative association between diastolic blood pressure and NO-response in controls (figure 4), while this was not statistically significant in ESRD. This further suggests a greater role of high blood pressure in the attenuation of endothelial function in resistance arteries than the uremia per se. The similar ADMA levels in patients and controls additionally suggest a relative low exposure to the uremic toxicity indicating well treated dialysis patients. This contrasts with the patients used in the previous studies [12], [13] where ADMA levels were significantly increased.

Interestingly we found a positive correlation between pulse pressure and micro-vascular vasodilatation in ESRD patients, which was not present in control subjects. This indicates that higher pulse pressure, which could translate into arterial stiffness, generates a process in the microcirculation that increases the vasodilator properties. Knowing that our patients are well treated in terms of blood pressure and with exposure to uremic toxins, we can speculate, that with longer exposure to renal deficiency, this compensation reaches its maximum and instead diminishing of the vasodilatation starts, as shown in other studies [11]–[13].

As expected the pulse wave velocity was increased in the ESRD population [20] – although in our study not statistically significant. At the same time cardiac output was higher possibly as a consequence of a reduced hemoglobin level.

Interestingly, we showed that the EDH-like response in healthy controls is dependent on the S-phosphate level, with a decreased vasodilator response with higher S-phosphate, even within the normal range of S-phosphate. We believe that further studies in this field are required to finally establish this correlation. Interestingly is has recently been shown that incubating resistance vessels from both healthy controls and patients with chronic kidney disease with a higher concentration of phosphate decrease the endothelium dependent vasodilatation [21]. The same group has shown that loading healthy people with phosphate reduces flow-mediated dilatation, indicating impaired endothelial function. After loading, S-phosphate was still within normal range [21]. Other studies have shown that FGF23, which might be an indicator of dietary phosphate load [22] is related to a reduced flow-mediated dilatation [23]. Our data indicates that the level of S-phosphate might affect the vasorelaxation.

When assessing cardiac output and sensitivity of resistance arteries to ACh, opposing associations were seen in controls and ESRD (figure 8). This could suggest that in healthy subjects, better vasodilator capacity in the resistance arteries is associated with a lower cardiac output, which could translate into diminished workload on the heart in the long term, and subsequent a lower risk of cardiac failure.

### Study limitation

The sample size of study groups was relatively small which possess a risk of committing type II errors and overinterpretation of the correlations. The majority of patients was receiving kidney transplants, and was therefore with less comorbidity than most patients in dialysis. This could explain why we see little difference between healthy controls and kidney patients in some of our assays.

### Conclusions

In conclusion this study finds similar vasodilator properties in small resistance arteries of ESRD and control subjects. ESRD patients were well controlled in terms of blood pressure and uremic exposure. However correlations of pulse pressure, diastolic blood pressure and cardiac output with micro-vascular functions, indicate compensating measures in the microcirculation of ESRD patients.
